# Galaxy tools to study genome diversity

**DOI:** 10.1186/2047-217X-2-17

**Published:** 2013-12-30

**Authors:** Oscar C Bedoya-Reina, Aakrosh Ratan, Richard Burhans, Hie Lim Kim, Belinda Giardine, Cathy Riemer, Qunhua Li, Thomas L Olson, Thomas P Loughran, Bridgett M vonHoldt, George H Perry, Stephan C Schuster, Webb Miller

**Affiliations:** 1 Center for Comparative Genomics and Bioinformatics, Pennsylvania State University, University Park, PA 16802, USA; 2 Terreus Foundation, 3938 Trust Way, Hayward, CA 94545, USA; 3 Penn State Hershey Cancer Institute, Hershey, PA 17033, USA; 4 Department of Ecology and Evolutionary Biology, Princeton University, Princeton, NJ 08544, USA; 5 Department of Anthropology, Pennsylvania State University, University Park, PA 16802, USA

**Keywords:** Genome analysis, Species conservation, Population structure, Selective sweeps, Admixture

## Abstract

**Background:**

Intra-species genetic variation can be used to investigate population structure, selection, and gene flow in non-model vertebrates; and due to the plummeting costs for genome sequencing, it is now possible for small labs to obtain full-genome variation data from their species of interest. However, those labs may not have easy access to, and familiarity with, computational tools to analyze those data.

**Results:**

We have created a suite of tools for the Galaxy web server aimed at handling nucleotide and amino-acid polymorphisms discovered by full-genome sequencing of several individuals of the same species, or using a SNP genotyping microarray. In addition to providing user-friendly tools, a main goal is to make published analyses reproducible. While most of the examples discussed in this paper deal with nuclear-genome diversity in non-human vertebrates, we also illustrate the application of the tools to fungal genomes, human biomedical data, and mitochondrial sequences.

**Conclusions:**

This project illustrates that a small group can design, implement, test, document, and distribute a Galaxy tool collection to meet the needs of a particular community of biologists.

## Background

A remarkable decrease in the cost of high-throughput sequencing has prompted many groups to address their biological questions by applying this technology. However, as is widely recognized, data analysis remains challenging for all but the largest and most experienced groups. Frequently, one of the challenges in this analysis is identifying the polymorphisms within species from the vast amount of raw data produced by the sequencing instruments, which requires computational resources and expertise not always available to a small biology lab. Fortunately, this and other data-processing steps are becoming more affordable as technology evolves.

Once the gigabases of sequence data have been filtered to, say, a few million intra-species DNA polymorphisms and a few thousand amino-acid variants, the computational requirements for their exploration are often relatively modest. Nevertheless, the analyses performed on the polymorphisms frequently require the expertise of the lab that originated and processed the biological samples, and it is burdensome for a small group to assemble and master the required computational tools. We have written a set of tools that run on the Galaxy web server to perform such analyses. They have an emphasis on understanding the population structure of the focus species and/or developing testable hypotheses about phenotypic consequences of genetic polymorphisms. In addition to avoiding the need for research groups to download and install all relevant software, a major benefit of using Galaxy for these, or indeed other, analyses is that reproducibility of published results is often enhanced. Moreover, these tools can be applied to polymorphisms identified by technologies other than sequencing, such as a SNP genotyping microarray.

The goal of this paper is to describe our Galaxy tools and illustrate their effectiveness. We begin with an overview of the new tools, after which we sketch how they were applied in a recently published manuscript, illustrate some of their capabilities, and compare their results with results published by other groups. These case-studies are listed in Table 1. Finally, we mention some lessons learned in the course of creating this Galaxy toolset.

**Table 1:** Examples discussed in this paper

**Species**	**Purpose of the case study**
1. Aye-aye	Tutorial on the new Galaxy tools
2. Chicken	Compare our selective-sweep analysis with published results
3. Canids	Compare our admixture analysis with published results
4. Human	Compare our admixture approach to other methods
5. Pigs	Illustrate the interplay among admixture and other analyses
6. Chytrid fungus	Apply our tools to non-vertebrates
7. Human	Apply our tools to biomedical data
8. Colugo, cave bear	Apply our tools to mitochondrial data

## Data description

Our tools work on polymorphism data in tabular formats that are appropriate for loading into the Galaxy web server [1-4]. The files for amino-acid variants and genes are basically just tab-delimited tables as required by Galaxy’s rich arsenal of table-manipulation tools. However, our single nucleotide variant (SNV) tables (which covers both intra-species SNPs and inter-species differences) have particular formats required by many of the tools that we have recently added to Galaxy, and a little familiarity with those formats is assumed in some of our later discussions.

**Figure 1:**
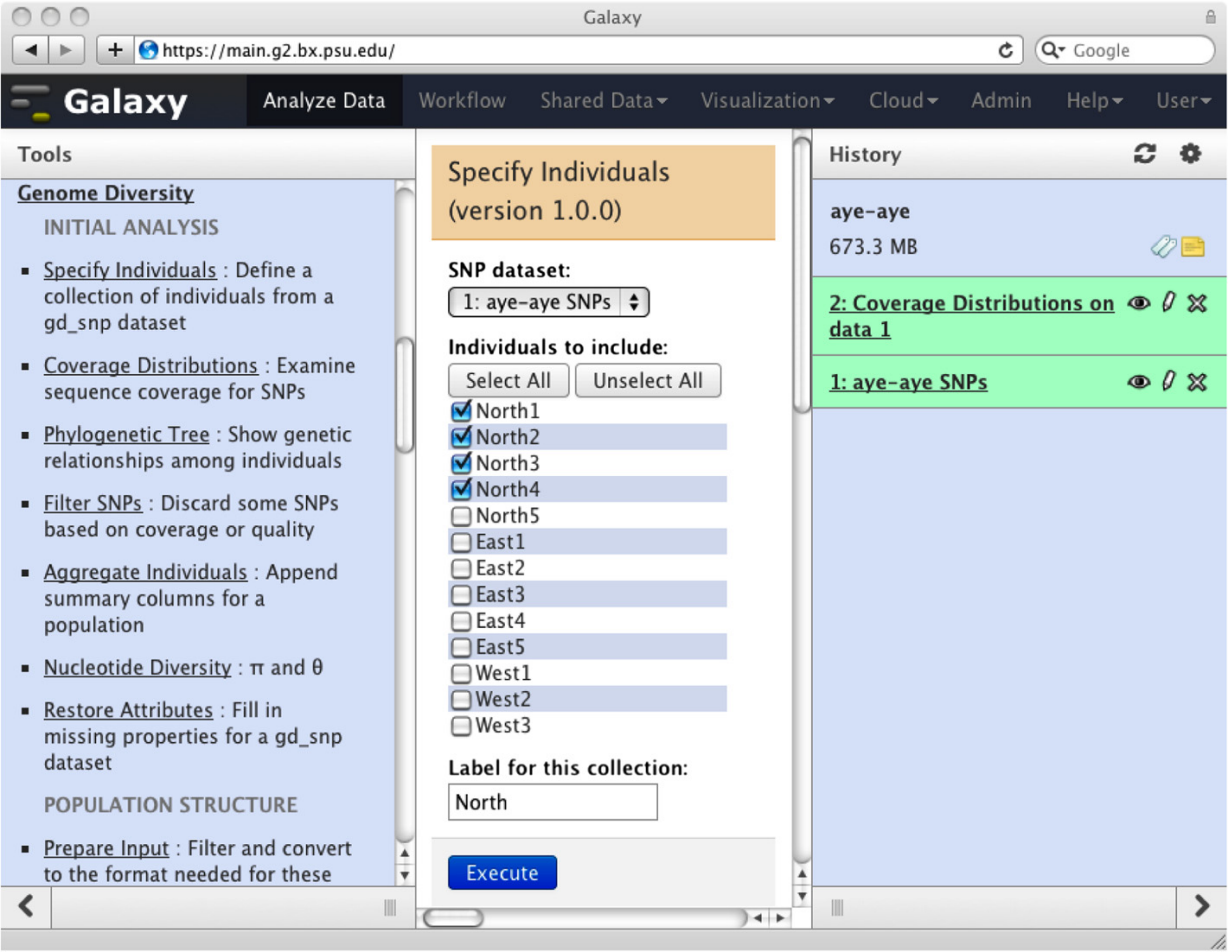
**Specifying a “population”.** The user is telling Galaxy that the individuals called “North1” through “North4” are to be considered a population called “North” in subsequent analyses (see the aye-aye example). Galaxy tools then know which columns of the SNV table to consult to locate information for further analysis.

Our most flexible format for SNV tables, called gd_snp (“gd” for Genome Diversity), has one row per SNV, and designated columns for the chromosome (and/or scaffold) name and position, the reference (or consensus) nucleotide, the variant nucleotide, and a quality value. For each individual (or sample) there are four columns, giving (1) the number of reads with the reference nucleotide, (2) the number of reads with the variant, (3) a genotype (0, 1, or 2 occurrences of the reference nucleotide; –1 = no genotype) and (4) a quality value for the genotype. A description of how columns are to be interpreted is specified in header lines, which can be prepared using one of our tools (#1 and 2 in the list below). Among other uses, this information lets Galaxy present the user with a simple interface for defining a set of individuals (Figure 1). In addition, there can be other columns, either supplied in the original table or generated by running Galaxy tools (e.g., each SNV’s *F*_ST_ value relative to two specified populations).

Galaxy also supports a simpler format, called gd_genotype, which differs from gd_snp by having just a single column per individual, giving the genotype. The overall quality value (column 5 for gd_snp format) can also be omitted and/or other columns can be included. We provide a Galaxy tool to facilitate the conversion of VCF files and three commonly used population genetics formats (i.e., FSTAT, Genepop, and CSV, see #2 in the tool list below). VCF files that include the field’s allelic depth, genotype quality and genotype (“AD”, “GQ”, and “GT” respectively in the “FORMAT” field) can be converted into a gd_snp file. We also provide a Galaxy tool to convert gd_snp and gd_genotype tables into a form suitable for submission to dbSNP.

The following is a list of the tools we have made available on Galaxy, under the heading “Genome Diversity”. This is the status as of November 2013; we expect that the list will grow over time.

A. *Basic Analyses*

1. *Make File: Create a gd_snp or gd_genotype file*

1. *Convert: Change CSV, FSTAT, Genepop or VCF to either gd_snp or gd_genotype*

1. *Specify Individuals: Define a collection of individuals from a SNV dataset*

1. *Coverage Distributions: Examine sequence coverage for SNVs*

1. *Phylogenetic Tree: Build a distance-based tree.*

1. *Filter SNPs: Discard some SNVs based on coverage or quality*

1. *Aggregate Individuals: Append summary columns for a population*

1. *Nucleotide Diversity: π and θ*

1. *Restore Attributes: Update properties of a SNV table*

1. *SNV table to VCF: For submission to dbSNP*

B. *Population Structure*

11. *Prepare Input: Filter and convert to the format needed for tools #12-14*

11. *PCA: Principal Component Analysis of genotype data*

11. *Population Complexity: Evaluate possible numbers of ancestral populations*

11. *Ancestry: Characterize ancestries with respect to inferred ancestral populations*

11. *Admixture: Map genomic intervals resembling specified source populations*

C. *F*_ST_*and Selective Sweeps*

16. *Overall F*_ST_*: Estimate the relative fixation index between two populations*

16. *Per-SNP F*_ST_*: Compute a fixation index score for each SNV*

16. *Remarkable Intervals: Find high-scoring runs of SNVs*

D. *Kinship*

19. *Close relatives: Discover familial relationships*

19. *Pairs sequenced: Offspring estimated heterozygosity of sequenced pairs*

19. *Founders sequenced: Estimated heterozygosity from a pedigree with sequenced founders*

19. *Matings: Assignment of optimal breeding pairs*

19. *Inbreeding and kinship: Analyze the pedigree without genomic data*

E. *KEGG and GO*

24. *Get Pathways: Look up KEGG pathways for given Ensembl transcripts*

24. *Rank Pathways: Assess the impact of gene sets on pathways*

24. *Pathway Image: Draw a KEGG pathway, highlighting specified gene modules*

24. *Rank Terms: Assess the enrichment of gene sets on Gene Ontology terms*

24. *Cluster KEGG: Group gene categories by shared genes*

F. *Design Genotyping Studies*

29. *Sample SNPs: Select a specified number of uniformly spaced SNVs*

29. *Differential Cleavage: Select SNVs differentially cut by specified restriction enzymes*

29. *Flanking Sequence: Fetch DNA sequence for intervals surrounding the given SNVs*

29. *Pick Primers: Find suitable PCR primers for SNVs*

G. *Mitochondria*

33. *Draw variants: show positions of SNVs and unsequenced intervals*

33. *Reorder individuals: exchange rows in the picture created by tool #33*

33. *Diversity: pi, allowing for unsequenced intervals*

33. *Phylip: prepare data for phylogenetic analysis by tool #37*

33. *RAxML: maximum-likelihood phylogenetic tree*

Many of these capabilities are familiar to biologists who analyze genome sequences or genotype genetic markers in population samples. However, some detailed understanding of tools #17 and #18 is assumed in our subsequent discussions. First, the *F*_ST_, or “fixation index”, assigns a value to each SNV based on the allele-frequency difference between two populations. Tool #17 (as well as #16) lets the user choose among the original definition of *F*_ST_[5] and two “unbiased estimates” of it [6,7]. The Reich formulation [7] has been shown to work well with small population sizes [8], and we use it exclusively unless stated otherwise.

Second, genomic intervals having particular characteristics, such as showing signs of a “selective sweep”, can be identified with the use of multi-individual sequence data. These sweeps are caused when an advantageous allele and neighboring linked variants increase their frequency in a population. Large *F*_ST_ values are one potential signature of a past selective sweep [9], though care must be taken because large *F*_ST_ values can also be created by genetic drift, demographic effects, or admixture [10-12]. We currently do not provide a tool for the traditional approach of scanning for unusual genomic intervals using a fixed “window” size, because in our opinion it would involve an undesirable number of user-selected values and options (e.g., specifying the window size, the amount of overlap between successive windows, the treatment of overlapping “significant” windows, discarding windows containing too few SNVs, etc.). Instead, we provide a tool (#18) that works with any chosen numerical column in a SNV table, for example, the *F*_ST_ relative to two populations or a value measuring homozygosity within a population. This tool has a single “tuning parameter”, which we call the “shift value”, set by the user. The tool subtracts this number from each SNV score, and then finds “maximal” intervals where the sum of shifted scores cannot be increased by adding or subtracting SNVs at the ends of the intervals. For instance, if the column in question contains *F*_ST_ values, the user could set the shift value at, say, the 90th percentile, so that 90% of the shifted values would be negative, and hence the SNVs in any high-scoring interval on average lie in the top 10%. In general, raising the shift value will lead to identification of fewer and shorter intervals. Statistical significance is estimated by a randomization strategy, in which the shifted SNV scores are shuffled some specified number of times, the highest-scoring interval found in each case, and the highest observed score is taken as the cutoff; this provides an empirical *p*-value, assuming that the scores are independent.

Another tool implementing a new algorithm estimates admixture in each of a set of individuals relative to two or three assumed source (often called “ancestral”) populations (tool #15). Roughly the same capability is available from other tools, such as SABER [13], HAPMIX [14] and PCAdmix [15]. However, we implemented a simpler approach for data sets that have inadequate numbers of individuals, inadequate accuracy of genotype calls, or uncertainty about basic species parameters (e.g., mutation rate and extent of linkage disequilibrium) to justify use of complex evolutionary models. This has the advantages of much shorter execution times and simplicity of use. Like tool #18, there is a single “tuning parameter”, which we call the “genotype switch penalty”.

With two source populations, the tool’s goal is to partition every autosome of an admixed individual into three inferred “interval genotypes”: (1) both chromosomes from the first source population, (2) both chromosomes from the second source population, or (3) one chromosome from each source population. (There are six interval genotypes in the case of three source populations). Suppose for a moment that a genomic interval is entirely in one of those categories, and we want to determine which interval genotype is most likely. For each SNP, we are given the observed genotype for the potentially admixed individual and can estimate the reference allele frequency in each source population. From those data, we can compute the probability of the observed sequence of SNV genotypes being produced from each of the interval genotypes, preferring the scenario with highest probability. The remaining issue concerns the choice of when to switch from one interval genotype to another, which is solved efficiently with a technique called “dynamic programming”, a relative of a partitioning method used with hidden Markov models. As the program scans along a chromosome, a larger genotype switch penalty makes it more difficult to switch between inferred interval genotypes, so the autosomes are partitioned into fewer, but longer runs of constant genotype.

We use this capability for exploratory data analysis, where we experiment with various thresholds on minimal spacing between SNVs (to increase independence), minimal *F*_ST_ between the source populations (to identify “ancestry informative markers”), and switch penalty, to reach conclusions that are robust to changes in analysis parameters.

In the following paragraphs we turn to a set of examples aimed at providing the reader with a clearer understanding of the range of capabilities of the Galaxy tools. Most of them deal with analyzing variation in the nuclear genome of non-model vertebrates, but one example studies polymorphisms in the fungus *Batrachochytrium dendrobatidis*, two examples consider human data, including differences between sequences from normal and LGL-leukemia diseased cells of the same individual, and one study of which, deals with mitochondrial sequence data.

## Analyses

### 1. Aye-aye

An analysis of low-coverage sequence data (roughly 5× coverage per individual) for aye-aye SNPs has been published [16] based on a *de novo* assembly of the aye-aye genome [17]. The aye-aye is a lemur species with a relatively wide geographic distribution around the periphery of Madagascar. The goal of our study was to assess the species’ genetic diversity and population structure, and relate these values to the geographical range. One possible outcome of this kind of investigation might be to identify sub-populations that warrant treatment as separate “conservation units” because of their genetic distinctiveness.

**Figure 2:**
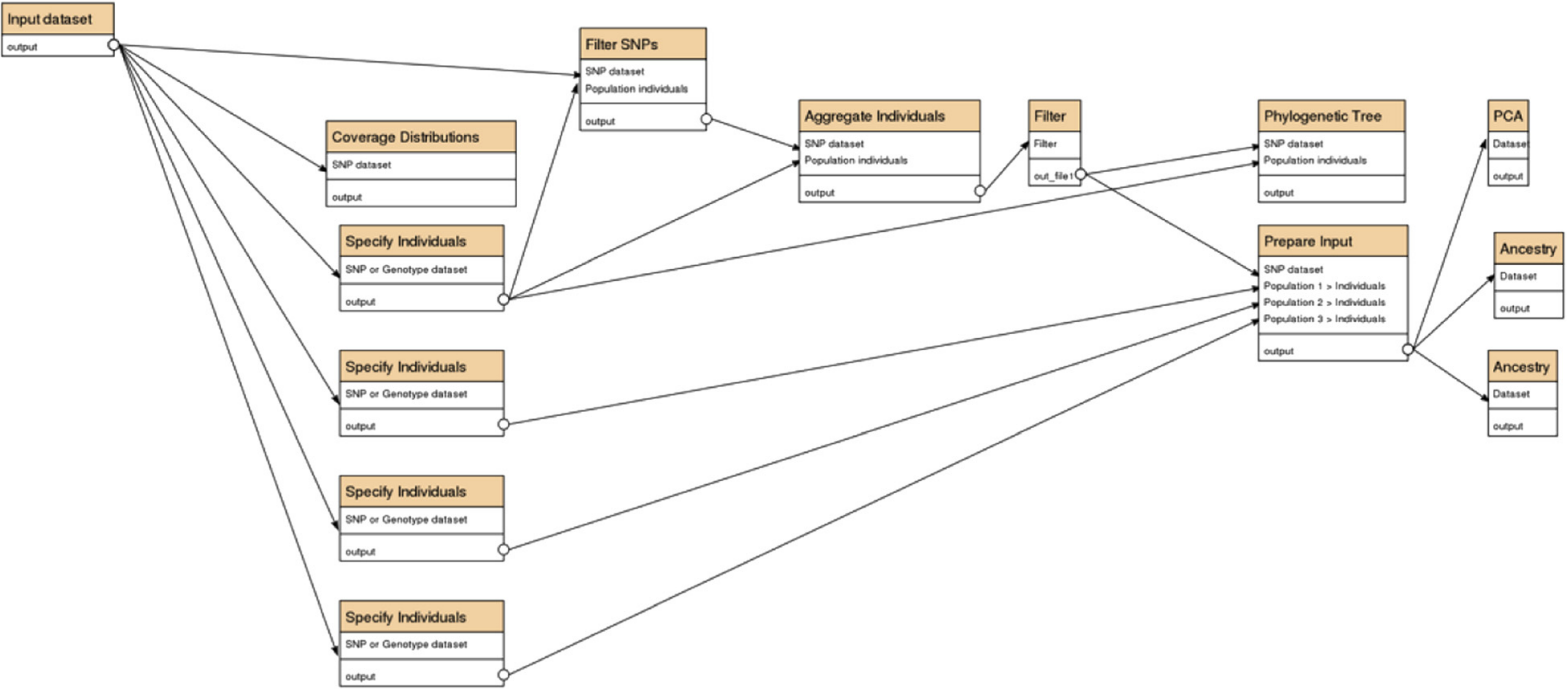
**Commands for the aye-aye example.** Depiction of the Galaxy commands needed to determine levels of sequence coverage, define sets of individuals, filter SNPs by depth of sequence coverage and non-fixation, draw a phylogenetic tree, perform a principal-components analysis, and analyze with 2 and 3 putative ancestral populations, as discussed for our aye-aye data.

We sequenced five individuals from the north of Madagascar, five from the east, and three from the west. A Galaxy coverage plot (tool #4 in the list above) showed that one of the samples from the north had particularly low sequence coverage, so we excluded it from further analysis. Also, for many SNPs the depth of sequence coverage seemed inadequate to support reliable estimations of genotype, so we omitted those SNPs from subsequent analyses (via tool #6), and also discarded SNPs where the coverage was so high as to suggest the presence of a regional duplication. The Galaxy phylogenetic-tree (#5) and PCA (#11, 12) tools indicated a clear population structure (further supported by tool #14), which appeared at first to be unrelated to the putative geographical source of the samples. We hypothesized that some samples had been mislabeled during handling, which we verified using PCR and Sanger sequencing experiments on separate DNA extractions from the source tissues, using primers identified by Galaxy tool #32 to amplify over a subset of the genotyped SNPs. We then specified three populations: North, West and East (tool #3; Figure 1). The phylogenetic tree, principal components and population-structure tools (#5, 11, 12, 14), then painted a consistent picture that the North population was particularly distinct. Figure 2 depicts the Galaxy commands that perform these analyses.

The Galaxy tools also provided estimations of within-group diversity, π (tool #8), and overall *F*_ST_ values for pairs of aye-aye populations (tool #16), which we evaluated by synthesizing a human data set that matched the aye-aye sequences in numbers of individuals and sequence depth. We found that the overall *F*_ST_ between the North and East populations appeared to be 2.1 times greater than that between human sub-Saharan Africans and Europeans, despite the fact that nucleotide diversity (tool #8) within each of the three aye-aye populations is relatively low.

In addition to SNV tables, our tools produce Galaxy tables of putative amino-acid polymorphisms. For the aye-aye, we mapped the assembled contigs [17] and the SNPs they contain to the human genome, and used human gene annotations to infer coding exons in the aye-aye. The results of that analysis have not been published, and we sketch some observations here to illustrate the use of additional Galaxy tools.

We calculated a Locus Specific Branch Length (LSBL) score for each SNP in each of the three aye-aye populations. LSBL is a function of the pairwise *F*_ST_ between populations, and helps to isolate the direction of allele frequency change. It has been extensively used in previous papers (e.g., [18]). We then selected the SNPs that mapped to coding regions and had an LSBL score in the top 5% for each population (i.e., LSBL95, with thresholds 0.6112 for North, 0.4365 for East, and 0.5536 for West). The LSBL score can be computed for each lineage using

$$\begin{array}{ll} \text{LSBL}\left(\text{North}\right)= & \left(\left[\text{North},\text{East}\right]+\left[\text{North},\text{West}\right]\right)\\ & \left(–\left[\text{East},\text{West}\right]\right)/2\;\text{and}\;\text{similarly}\;\text{for}\\ & \;\text{LSBL}\left(\text{East}\right)\;\text{and}\;\text{LBSL}\left(\text{West}\right). \end{array}$$

**Figure 3:**
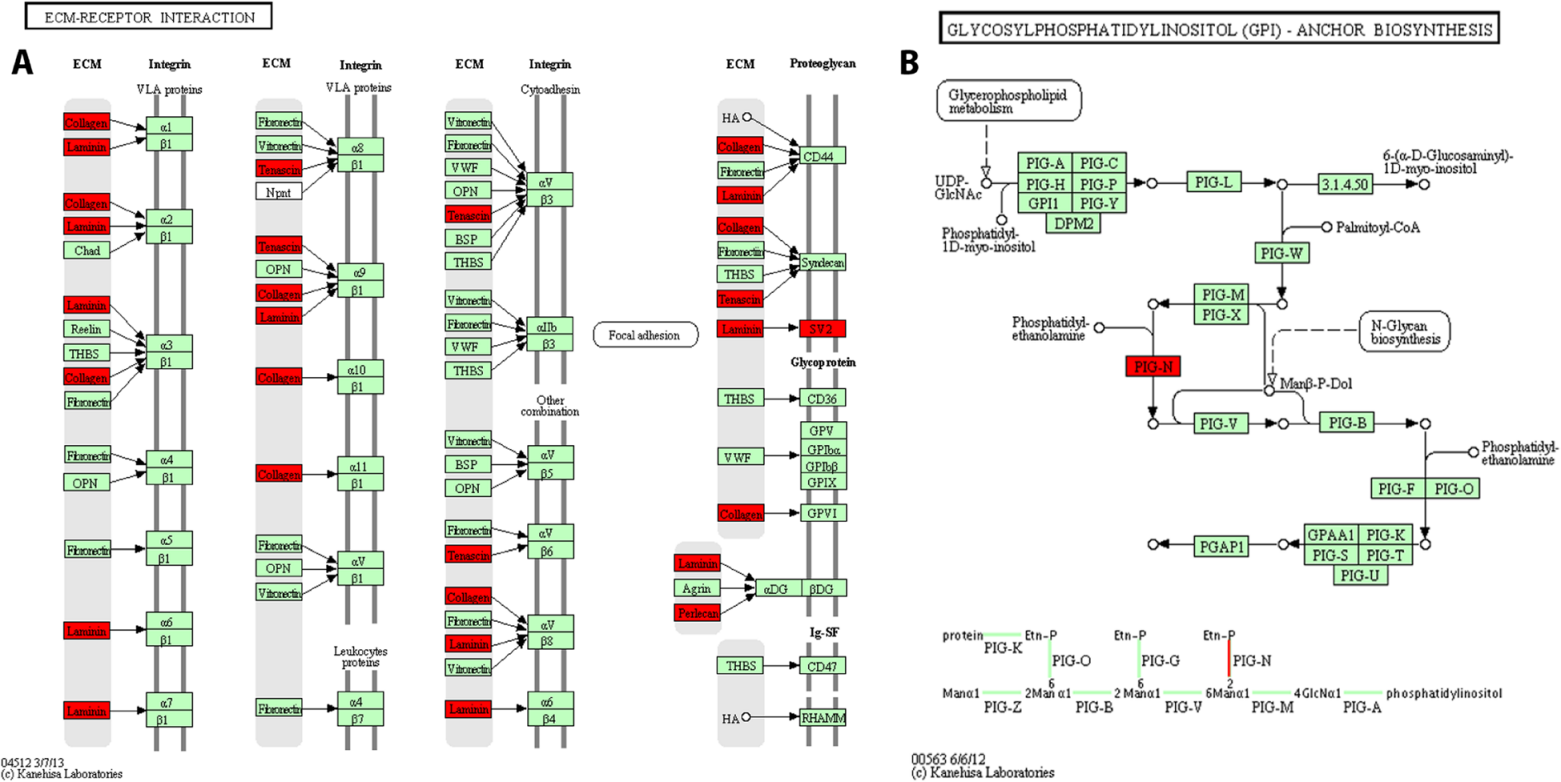
**Two KEGG pathways from the aye-aye data. A)** KEGG pathway diagram showing the genes with coding mutations involved in the extracellular matrix-receptor interaction pathway. Eleven genes with SNPs in the top 5% by LSBL score in the West aye-aye population appear in this pathway, including three with non-synonymous mutations (*LAMC2, HSPG2*, and *LAMA3*). These genes are grouped in 5 different functional units distributed along the pathway (i.e., collagen, laminin, tenascin, perlecan, and SV2, all shown in red). **B)** KEGG pathway diagram for the Glycosylphosphatidylinositol-anchor biosynthesis pathway showing the central role of the *PIG-N* gene for GPI-anchor synthesis.

1. For each pair of populations, calculate the pair’s *F*_ST_ value for each SNP (using tool #17).

2. Use the standard Galaxy tool called “Compute an expression for every row” to compute, for each SNP:

We identified 390 coding mutations in the North population, 373 in the East and 420 in the West (above the LSBL95). Of these, the number of non-synonymous SNPs was roughly the same in the three populations (150 in 129 genes for North, 133 in 121 genes for East, and 134 in 128 genes for West). We looked for Kyoto Encyclopedia of Genes and Genomes (KEGG) pathways in which these genes are known to be involved using the Get Pathways tool (#24), and then ranked them by percentage of genes affected using the Rank Pathways tool (#25). For this discussion, we consider only the West aye-aye population, for which this tool produced a list of 153 KEGG pathways for the genes with synonymous mutations, and 83 for the genes with non-synonymous mutations. For instance, the extracellular matrix (ECM) receptor interaction pathway was placed second in the synonymous ranking and third in the non-synonymous ranking. This pathway was one of eleven significantly enriched pathways for genes in the synonymous list (p = 3.8 × 10^-7^), and one of four in the non-synonymous list (p = 0.018). Three genes with non-synonymous mutations (*LAMC2, HSPG2*, and *LAMA3*) and eight with synonymous mutations (COL4A2, COL5A1, LAMA4, LAMB1, LAMB4, LAMC1, TNN, and SV2B) are associated with this KEGG pathway. We used the Pathway Image tool (#26) to visualize the genes’ roles in the pathway (Figure 3A).

In support of these results, the Rank Terms tool (#27) produced a list of GO terms related to ECM-receptor interaction that were significantly enriched in the genes with non-synonymous mutations above LSBL95. These GO terms included “cytoskeletal anchoring at nuclear membrane” (*p* = 4.6 × 10^-5^), “laminin-5 complex” (*p* = 1.4 × 10^-4^), “basement membrane” (*p* = 0.0016), and “cell adhesion” (*p* = 0.0067). Also, we grouped the GO terms and KEGG pathways with non-synonymous mutations in the West population using the Cluster gene categories tool (#28). We used different thresholds to study the groups produced and found at a cutting threshold of 20% a cluster of gene categories that include the terms “response to glucose stimulus”, “collagen type VI”, “muscle organ development”, “sarcolema”, “extracellular matrix structural constituent”, “focal adhesion”, and “PI3k-Akt signal pathway”. Furthermore, we found consistently (with thresholds ranging from 25% to 85%) the cluster of gene categories: “response to glucose stimulus”, “collagen type VI”, “muscle organ development”, and “sarcolema”.

We studied the potential effects of non-synonymous mutations in the West population by ranking the KEGG pathways according to the changes in length and number of paths if the genes are disrupted. Among the five KEGG pathways that showed changes in both of these values, the Glycosylphosphatidylinositol (GPI)-anchor biosynthesis pathway was ranked first (change in the mean length of paths between sources and sinks = 4.5, change in the number of paths between sources and sinks = 4). The image of this pathway (Figure 3B; produced using tool #26) shows that a mutation in the gene *PIG-N* could disrupt the transference of phosphatidylethanolamine to the first mannose of the glycosylphosphatidylinositol. This result revealed a picture that could not have been obtained by using the overrepresentation approach: despite that only one gene (out of 23) was found to have a non-synonymous mutation, the role of this gene is required and critical in the GPI-anchor biosynthesis. Genes involved in both extracellular matrix-receptor interactions and cell adhesion (including GPI-anchor production) are implicated in tissue morphogenesis and organization [19,20]. Their role has been described in the organogenesis of kidney, lung, peripheral nerves, brain, extremities, digits, pancreas and placenta, as well as in the integrity maintenance of skeletal muscles, skin, and hair [20]. The modules laminin and perlecan in the ECM-receptor interaction pathway include genes with non-synonymous mutations (*LAMC2*, *HSPG2*, and *LAMA3*). Both of these modules are involved in the linkage of extracellular matrix with dystrophin through dystrophin-associated glycoproteins (alpha-DG and beta-DG in Figure 3A; [21]). A failure in this linkage has been extensively associated with muscular dystrophy, as dystrophin is thought to provide mechanical reinforcement to the sarcolemma to protect it from the membrane stresses developed during muscle contraction [21-23].

The mutations affecting matrix-receptor interactions and cell adhesion are expected to evolve in concert as organisms adapt to specific niches [19,24]. Aye-ayes are highly specialized extractive foragers; they feed on insect larvae obtained from decaying tree bark, and on seeds. It has been suggested that limitations in the availability of food may explain the large individual home range requirements of this species [17]. Previous papers have reported a relatively complex neuromuscular organization for lemurs, and have proposed that this is consistent with differences in habitat and surface utilization (e.g., arboreal vs. ground) [25,26]. Additionally, a potential for increased stress on the aye-aye's long gracile digits is generated during its locomotion, especially while descending trees [27]. It is difficult to assess the extent to which the molecular mechanisms reported here may be implicated any kind of ongoing adaptation among aye-aye populations. However, one interpretation is that they might be involved in muscular adaptations to exploit the niche variability produced by the landscape variation, habitat diversity, and microendemism patterns of northern Madagascar [28]. This example illustrates the use of some of our new tools, as well as the kinds of hypotheses they can lead to.

### 2. Chicken

A number of methods have been developed for detecting evidence of selective sweeps using polymorphism data from multiple individuals, with each method exploiting a particular departure from the expectation with neutral evolution [29]. A typical application of these methods is to identify genomic regions related to reproductive fitness, such as those conferring traits important for adaptation to a new environment. Several tools to support such analyses can be found in the new Genome Diversity toolset on Galaxy, and we wanted to compare their performance with accepted techniques.

The chicken genome was one of the first vertebrate genomes to be published [30]. An analysis of multi-individual data was published later [31], where a windows-based approach was used to look for regions of low heterozygosity in various combinations of domestic breeds, with the goal of identifying genomic regions associated with economically important traits, such as egg or meat production. We were interested in understanding how much, and under what conditions, their results differ from genomic intervals found by our windows-free method.

The published project [31] sequenced ten samples from different chicken breeds, nine of which were each a pool of DNA from several individuals. Their analysis was carried out on the numbers of reads corresponding to the more common and less common allele, whose values were calculated for each combination of SNV and DNA sample. The authors kindly provided us with those numbers, from which we produced a Galaxy SNV table (gd_snp format) with 7,285,024 rows (i.e., SNVs) and 45 columns (see Methods).

**Figure 4:**
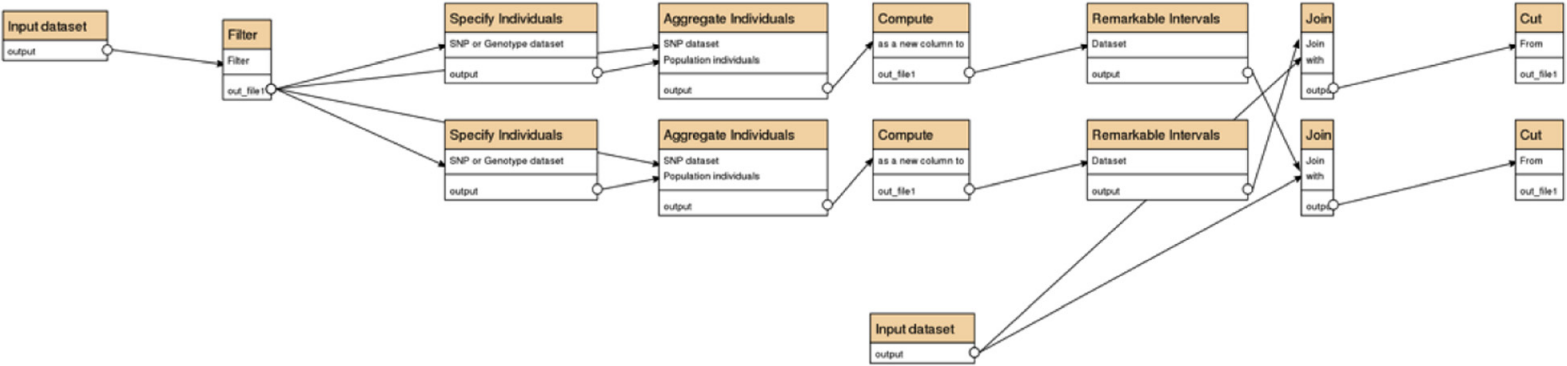
**Commands for the chicken example.** Depiction of the Galaxy commands for finding genes contained in autosomal intervals of unusually high heterozygosity in eight pooled populations of domestic chickens and in two pooled samples of domestic broilers. The input datasets are the set of 7.3 million SNVs and a list of chicken genes with their chromosomal coordinates.

A search for regions of high homozygosity and the genes within them can be conducted, starting with the SNV table and a list of chicken genes, by the following Galaxy commands, which are also depicted in Figure 4.

1. Specify individuals (tool #3 listed above), for example, all pools from domestic chickens, or all Commercial Broilers.

2. Aggregate those individuals (tool #7), to get totals of the reference alleles (column 46) and the variant alleles (column 47).

3. Use a standard Galaxy tool to compute (into column 50) the expression

$$\left(c46*c46+c47*c47\right)/\left(\left(c46+c47\right)*\left(c46+c47\right)\right)$$

where c46 and c47 are the values in columns 46 and 47. Intuitively, the two allele frequencies are c46/tot and c47/tot, where tot = c46 + c47, and we are adding their squares to quantify homozygosity.

4. Use the Remarkable Intervals tool (#18), setting the shift value to a desired threshold, say 0.9, to find intervals where the sum of the scores c50 – 0.9 is high; c50 is the value assigned to a SNV by step 3 (i.e., homozygosity).

5. Use a standard Galaxy tool to find genes that intersect the intervals identified by step 4.

For the pool, the AD of all domestic individuals, 158 intervals of average length (approximately 85 kb) were reported [31]. The intervals cover a total of 13.4 Mb, or approximately 1.3% of the chicken genome. We set the threshold in step 4 (see above) to 0.78, chosen by trial and error so that the average length of the 158 highest scoring intervals was also 85 kb. For the most part, the reported intervals agree with the highest-scoring intervals found by our window-free method. Our seventh highest-scoring interval, chr5 43,222,353-43,275,554, and their top-scoring segment, chr5 43,200,000-43,280,000, overlap the *TSHR* gene, which is a major focus of the paper [31]. Our twelfth and their fourth highest scoring interval (6,252,242-6,301,349 and 6,240,000-6,300,000 on chromosome 24, respectively) overlap the gene *BCDO2* for the yellow skin allele, which the authors of the original paper adopt as a proof of principle that a method can identify a known sweep [32]. In all, 89 of their regions overlap one of our 100 highest-scoring intervals.

For other measurements of concordance between the two approaches, consider regions of low heterozygosity in the two commercial broiler lines, which are bred for efficient meat production. The paper [31] identified 132 intervals of average length around 62 kb, while we used the threshold 0.9 in step 4 (see above) to get an average length around 64 kb (close enough) for our highest scoring 132 intervals. One of the top-scoring reported intervals, chr1 57,340,000-57,560,000, contains several genes related to growth, including insulin-like growth factor 1 (*IGF1*). In our approach, the interval chr1 57,356,555-57,574,111 scores highest. The other interval reported as under selection in commercial broilers is chr4 71,720,000-71,860,000, containing the *TBC1D1* gene, which had earlier been identified in several independent studies as the major Quantitative Trait Locus (QTL) explaining differences in growth between broilers and layers. Accordingly, our seventh highest-scoring interval is chr4 71,709,127-71,847,930, which also overlaps *TBC1D1*. Overall, our 100 highest-scoring intervals intersect 67 of their intervals. We also noticed a tendency for our highest-scoring intervals to overlap the 56% (74 of 132) of their intervals that intersect genes; our 20 highest scoring intervals overlap 15 of their gene-intersecting intervals, but only three of their intervals that do not intersect any annotated gene.

However, major differences between intervals found by the authors’ window-based approach and our window-free method can arise. Compared with our approach, their particular windows-based method favors regions with a low density of SNVs. Consider a simple example where one window has 10 SNVs, all fixed in the domestic birds (say n_Maj_Allele_ = 30 and n_Min_Allele_ = 0) and a second window with 100 of such SNVs. Then both windows score 0 according to the published approach, On the other hand, our approach instead works with homozygosity = 1 – heterozygosity, which is 1.0 for these SNVs. A threshold (for instance 0.9) is subtracted to give a score of 0.1, and the scores are added for each genomic interval, giving totals of 1.0 for the first interval (window) and 10.0 for the second, and a preference for the interval with more SNVs.

The seventeenth highest scoring reported interval for sweeps in commercial broilers [31], chr2 84,660,000-84,720,000, is not known to overlap any gene. The 1,272^nd^ best interval from our approach (far from being statistically significant) is chr2 84,662,385-84,719,725. It is possible that the main source of this discrepancy between the two methods is the extremely low number of SNVs at chr2 84,660,000-84,720,000, namely 31 SNVs in the 60 kb interval. Giving the nearly 7.3 million SNVs in the 1 Gb chicken genome, the expected number of SNVs in this interval is around 450, making the interval an extreme outlier. We believe it is counter-intuitive to consider genomic intervals with an extremely low density of SNVs as likely candidates for having experienced (or still experiencing) positive selection; low SNV density seems more indicative of negative selection.

### 3. Canids

The demographic history and relationships between lineages of North American *Canidae* has often been studied using a handful of genetic markers with limited resolution of evolutionary relationships. Specifically, a few dozen co-dominant or uniparentally-inherited markers will only provide a fraction of the evolutionary history. One of the main and long-debated topics of North American canids has been the degree of admixture and species ancestries. Answers to those questions could potentially influence conservation planning. For instance, if an endangered species is identified to have a significant degree of genetic admixture, then the management options become less obvious and the relevant conservation policy may, most likely, need to be updated. To best address admixture among canids and better resolve their ancestry, a published study [33] analyzed genotypes from 48,036 SNVs (hereafter, referred to as 48 K) distributed genome-wide. In order to test the robustness of our tools, we reanalyzed the same dataset for admixed ancestry across wolves and coyotes of North America.

**Figure 5:**
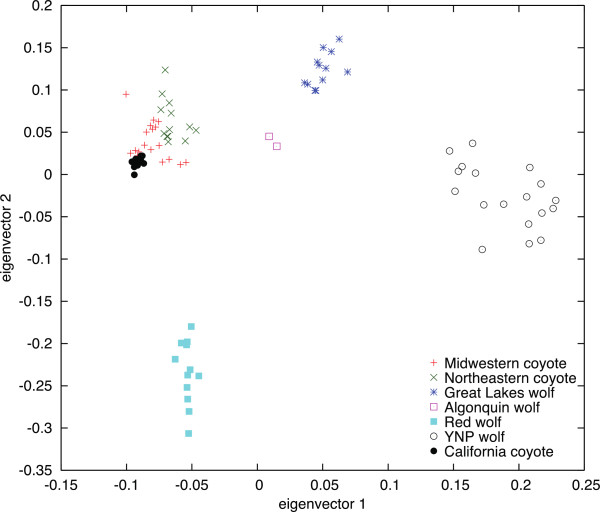
**Principal components analysis of canid data.** PCA of the reference (California coyote and YNP wolf) and putatively admixed North American canid populations using 48 K SNVs.

After formatting the 48 K data, individuals were designated into specific groups for subsequent testing. The California coyote (n = 12) and Yellowstone National Park gray wolves (YNP, n = 18) were labeled as non-admixed reference groups, with five putatively admixed groups identified for testing wolves from Algonquin Provincial Park (n = 2) and the Great Lakes region (n = 12), the Red wolf (n = 12), and two populations of coyotes (Northeastern, n = 13; Midwestern, n = 19) (Table 2A). To confirm the data, we conducted a principal component analysis (PCA) of the SNV genotypes and identified reference and admixed populations (Figure 5).

**Table 2:** Comparison of percentage of admixed ancestry results

**A.**					
	**2-ancestor model**		**3-ancestor model**		
**Putatively admixed group**	**Coyote**	**Wolf**	**Coyote**	**Wolf**	**Dog**
Red wolf (n = 12)	76.1 (144)	23.9 (184)	--	--	--
Algonquin wolf (n = 2)	41.9 (100)	58.1 (100)	--	--	--
Great Lakes wolf (n = 12)	14.9 (298)	85.1 (230)	--	--	--
Northeastern coyote (n = 13)	--	--	82.2 (96)	8.7 (51)	9.1 (16)
Midwestern coyote (n = 19)	--	--	90.1 (75)	2.4 (140)	7.5 (15)
**B.**					
**Switch value**	**Red wolf (n = 12)**	**Algonquin wolf (n = 2)**	**Great Lakes wolf (n = 12)**	**Northeastern coyote (n = 13)**	**Midwestern coyote (n = 19)**
	**2-ancestor model**			**3-ancestor model**	
0.5					
Coyote	66.9	52.8	41.9	71.2	76.4
Wolf	33.1	47.2	58.1	17.4	13.4
Dog	----	----	----	11.5	10.2
1.0					
Coyote	66.8	51.2	38.9	70.8	77.0
Wolf	33.2	48.8	61.1	17.4	12.9
Dog	----	----	----	11.7	10.2
3.0					
Coyote	71.0	47.3	30.1	73.2	82.3
Wolf	29.0	52.7	69.9	16.3	8.7
Dog	----	----	----	10.6	9.0
7.0					
Coyote	76.1	44.7	21.6	76.7	87.7
Wolf	23.9	55.3	78.4	14.0	4.4
Dog	----	----	----	9.3	7.9
10					
Coyote	78.8	40.5	18.1	78.1	89.2
Wolf	21.2	59.5	81.9	12.9	3.4
Dog	----	----	----	9.0	7.4
**C.**					
			**Coyote Ancestry Proportion:**		
***F***_***ST***_	**SNV Spacing**	**n**_**SNV**_	**Switch = 2**	**Switch = 5**	**Switch = 10**
Any	Any	48,036	67.8%	70.0%	71.3%
Any	≥ 0.5 Mb	3,838	67.7%	70.1%	71.9%
≥ 0.4	Any	7,875	69.4%	72.6%	75.4%
≥ 0.4	≥ 0.5 Mb	2,562	70.9%	76.2%	81.2%

**A)** Results from SABER [13], using the 2- or 3-ancestor model (initial admixture times, in generation units, are provided in parentheses from reference [33]) **B)** Results from Galaxy’s *Admixture* tool with various switch values; **C)** the variability of estimated average coyote percentage contributing towards the Red wolf genomes, depending on possible lower bounds imposed on *F*_ST_ and/or spacing between SNVs, as well as switch penalty (n_SNV_, number of SNVs).

From the published analysis [33], we had expectations *a priori* as to the ancestry composition of each individual. We began our two-ancestor dataset construction with computing per-SNP *F*_ST_ values (tool #17) using Wright’s original definition [5] to identify and retain 4,229 SNVs with *F*_ST_ > 0.4 using the two non-admixed reference populations of Yellowstone wolves and California coyotes (as per the published inclusion threshold [33]) as Ancestry Informative Markers (AIMs) for analysis using the admixture tool (#15). Next, we filtered SNVs with tool #6 to retain 3,175 SNVs with an average spacing of 1 SNV per 100 kb in order to construct a SNV set comparable to the published one [33]. These methods were repeated with the inclusion of a third possible ancestor population, domestic dogs (n = 28 from modern breeds). We conducted two additional per-SNP *F*_ST_ analyses to compare the YNP wolf and California coyotes each with the domestic dog. As a result, we therefore filtered to keep SNVs every 300 kb to retain similar marker counts (6,375 SNVs for 3-ancestor analysis). Finally, we conducted independent analyses for each of the putatively admixed populations (Table 2A).

As per the algorithm used by SABER [13] to estimate the time since initial admixture (e.g., length of blocks and recombination rate), there is a significant negative correlation between ancestry proportion and admixture times (*r* = -0.810, 1-tail *p* = 0.04819). When we surveyed the impact of varying the switch value of the Galaxy admixture tool (#15), it appears to be sensitive to the timing of initial admixture. All populations had a significant correlation between ancestry and switch values (Red wolf: *r* = 0.9914, 1-tail *p* = 0.0005; Algonquin wolf: *r* = -0.9809, 1-tail *p* = 0.002; Great Lakes wolf: *r* = -0.9716, 1-tail *p* = 0.003; Northeastern coyote: *r* = 0.9891, 1-tail *p* = 0.0007; Midwestern coyote: *r* = -0.9721, 1-tail *p* = 0.0028). The captive Red wolf, for example, has been extensively monitored in their recovery area, and recent coyote hybridization events have been documented [33-36]. As a result, coyote ancestry is expected to be elevated in the Red wolf genome. Varying the switch parameter, we found that larger values (switch ~ 7-10) estimated a level of coyote ancestry comparable to that with SABER (Table 2B,C). An analogous demography is suspected of the Northeastern coyote, with the population harboring many hybrids of recent ancestry [33]. With Galaxy, we found low levels of switch variation (mean ± SD coyote ancestry: 74% ± 3.3) and consistent representation of two ancestries within the Northeastern coyote genome (74% coyote, 26% gray wolf; Table 2B).

Admixture can result from highly complex demographic processes, either through mating preferences (e.g., [dis]assortative), backcrossing, selective removal of hybrids, or natural mechanisms of isolation. Regardless of the admixture process, quantifying ancestry is central for exploring population demography and conservation management efforts. Here we show that tabulating ancestry blocks across multiple canid genomes can be obtained quickly and accurately using the Galaxy admixture tool.

### 4. A southern African genome

Understanding admixture in humans is crucial for correctly tracing the flow of human populations, and it plays a substantial role in identifying genomic variants that affect disease susceptibility. Moreover, many people express a strong interest in knowing their ancestry. For instance, an individual, called ABT, of mostly Bantu-speaking heritage from South Africa, was thrilled when told that he also has some Khoisan (“bushman”) ancestry [37].

After that original discovery, we combined 419,974 microarray-based genotype calls for 485 humans, and added ABT’s genotypes inferred from whole-genome sequence data. Among the 485 individuals, 89 were identified as Yoruba (a well-studied population of agriculturalists from Nigeria) and 67 as Khoisan. We used this dataset to compare our admixture results (tool #15) with those obtained by the programs HAPMIX [14] PCAdmix [15] and ADMIXTURE [38,39]. With the Galaxy tool and PCAdmix, we used a third potential source population, CEU (certain individuals of European ancestry) and specified K = 4 ancestral populations with ADMIXTURE.

The programs gave reasonably comparable results: ABT’s Yoruba ancestry was estimated as 78.3%, 70.5%, 77.9% and 74.1% by HAPMIX, PCAdmix, ADMIXTURE, and the Galaxy tool, respectively. All of the programs of course depend somewhat on the settings chosen for input parameters; for instance HAPMIX produced values between 73.1% and 79% when we varied two parameters (i.e., number of generations since admixture and estimated proportions from the ancestral populations) over a wide range. PCAdmix appeared to have some problems with these data, leaving approximately 15% of ABT’s genome as having undetermined ancestry. Part of the reason that HAPMIX produces higher estimations of Yoruba admixture than the other tools is that it only allows two source populations. For example, limiting the Galaxy tool to two source populations (Khoisan and Yoruba) raises its estimation to 76.5% Yoruba.

### 5. Pig

The different breeds of domestic pigs are the result of a long domestication process from wild boars (*Sus scrofa*). During this process, qualities of importance to humans have been selected, shaping the genome landscape of the domestic breeds [40]. It is estimated that European and Asian wild boars split about 1 million years ago, with their domestication occurring independently on each continent [41]. Signals of positive selection have been found in domestic breeds that are associated with color, vertebrate number, and muscle development [40,41]. This exemplifies a number of studies that seek to identify genotype-phenotype relationships in domestic breeds, to potentially improve breeding practices, as with the chicken study, mentioned above.

We obtained 48,649,642 SNVs for 6 outgroup species and 49 *Sus scrofa* individuals (36 European, 6 Chinese, and several from other regions) used in those previous studies, and attempted to recapitulate some of the published results using the Galaxy tools. Following the approach of the chicken analysis, we calculated the homozygosity for four European breeds (n = 25 individuals), one Asian (n = 4) and one European wild boar (n = 6) breed [40, dataset 2]. The starting point for this analysis was a gd_genotype formatted file; thus, instead of using the aggregation tool (#7) we calculated the number of reference and alternative alleles for each population as follows:

1. Determine the columns with the genotypes of the individuals of interest (for the Asian breed c34, c35, c36 and c37).

2. Calculate the number of reference alleles in the individuals of interest (for the Asian breed ((c34==2)*2) + ((c35==2)*2) + ((c36==2)*2) + ((c37==2)*2) + ((c34==1)*1) + ((c35==1)*1) + ((c36==1)*1) + ((c37==1)*1)).

3. Calculate the number of alternative alleles in the individuals of interest (For the Asian breed ((c34==0)*2) + ((c35==0)*2) + ((c36==0)*2) + ((c37==0)*2) + ((c34==1)*1) + ((c35==1)*1) + ((c36==1)*1) + ((c37==1)*1)).

Further, we followed steps 3, 4, and 5 of the homozygosity calculation explained in the chicken example.

Published data [40] identified 70 selective sweeps genome-wide with a mean length of 878 kb. By trial and error we selected a shift score of 0.9889 for which the 70 top scoring intervals presented a mean size of 877 kb. Eleven of our 50 highest-scored intervals intersected reported selective sweeps [40], three of which overlapped the genes *NR6A1*, *PLAG1*, and *LCORL* to which the original study devotes a large discussion. The lengths of the intervals identified by our program agreed well with those reported previously. The lengths were on average 0.32 kb different, and exceeded 1 kb in only two cases. We attribute the observed differences to the limitations that a windows-based approach imposes: the resulting selective sweeps can only be as small or big as the specified window size. This limitation is illustrated by the intervals overlapping the gene *LCORL* (located in the chromosome 8 between 12,633,950 bp and 12,766,041 bp). While the windows-based approach found a sweep between 12,540,000 bp and 12,840,000; our windows-free approach determined that this interval was between the positions 12,555,236 bp and 12,807,451 bp. Thus, our approach better localizes the selective sweep to the *LCORL* gene, excluding a non-gene region between 12,807,452 and 12,840,000 bp.

To further explore the domestication dynamics in pigs, we used admixture studies. A PCA (with tool #12) indicated that European domestic swine, including the Landrace breed, are much closer to European wild boars than to Asian wild boars (also indicated by the phylogenetic-tree tool, #5), whereas an admixture analysis (with tool #15) suggests that about 10% of the Landrace genome is ultimately derived from Asian boars, mostly from southern China (possibly from intended crossings of European and Asian domestic swine). According to our analyses, genes overlapping the regions of Landrace genomes that have an origin in Chinese breeds are enriched for immune-related GO terms, including “defense response” (*p* = 8.7 × 10^-11^), “response to virus” (*p* = 5.6 × 10^-6^) and “defense response to bacterium” (*p* = 0.012).

The presence of this admixture would confound a search for selective sweeps based solely on the *F*_ST_ between a European domestic breed and European wild boars, because a genomic interval in the domestic breed that is derived from the Asian lineage will tend to have an *F*_ST_ that exceeds the genome average. Several approaches have been developed to circumvent this difficulty (e.g., [12]). A simple plan is to use the so-called LSBL [18], which treats the European and Asian wild boars symmetrically. Additionally, this score allows us to explore the specific selection occurring in the domestic breed lineage:

$$\text{LSBL}=\left(F_{\mathrm{ST}}\left(L,\mathrm{EW}\right)+F_{\mathrm{ST}}\left(L,\mathrm{AW}\right)–F_{\mathrm{ST}}\left(\mathrm{EW},\mathrm{AW}\right)\right)/2$$

where L = Landrace, EW = European wild boar, and AW = Asian wild boar.

After calculating LSBLs for each group and each SNP, we determined remarkable intervals with them (using tool #18). As previously suggested, we found that genes *NR6A1* and *PLAG1* overlap a potential selective sweep (further analyses reveal that these genes do not overlap intervals of high LSBL in European or Chinese breeds). These genes have been in QTLs associated with an increase in the number of vertebrae in domestic pigs and body height, respectively [40]. Other genes previously related to back fat thickness were also found to overlap intervals of high LSBL in Landrace, including *ALMS1*, *ACP2* and *ENPP1*[42]. Finally, one of those intervals overlaps the gene *VRTN*, previously found in a QTL suspected to cause heterogeneity of the number of vertebrae in commercial-breed pigs [43]. Other genes overlapping selective sweeps for the Landrace breed have been previously reported in QTLs of commercial interest for pig (i.e., *ELOVL6*). As with other studies [32,40], we assume that finding selective sweeps overlapping previously reported QTLs is a validation for our approach.

In the same line of inquiry, we determined regions that had been potentially under positive directional selection in the lineage leading to Landrace pigs. To do so, we selected SNVs with one variant fixed in the two closed related species *Sus barbatus* (Bornean bearded pig) and *Sus verrucosus* (Java warty pig) and the other fixed in the Landrace individuals. Further, we determined genes overlapping regions with a remarkable number of these markers, and studied their enrichment in GO terms and KEGG pathways (tools #25 and #27). One of these regions, which includes the genes *SPATA7* and *TTC8*, overlapped a QTL previous described for porcine intramuscular fat content [44]. 903 genes were found to overlap regions enriched in SNVs under potential positive directional selection, including *NR6A1* and *CASP10*. The *CASP10* gene has been found in a putative selective sweep with several duplications in domestic pigs [40]. Among the GO terms and KEGG pathways we found that "skeletal system morphogenesis" was significantly enriched for genes overlapping these regions (*p* = 0.0037).

In summary, using Galaxy tools, with special attention to the possible confounding of selective-sweep analyses by the presence of admixture, we were able to recapitulate published results and highlight additional genes of potential commercial interest.

### 6. Chytrid fungus

The chytridiomycota *Batrachochytrium dendrobatidis* (Bd) has been linked to the global decline of amphibians [45,46]. To shed light on the evolutionary history of this pathogen and to identify genomic underpinnings of its virulence, a recent paper reported the genomes of 29 Bd isolates from around the world [46]. Among other results, the authors analyzed genes potentially under positive selection (*d*_N_ > *d*_S_), those in regions exhibiting loss of heterozygosity (LOH), and those in regions with copy number variations (CNV), focusing on strains in the Global Panzootic Lineage (GPL) associated with the disease. This study illustrates the use of genome sequencing to uncover the evolutionary history of an emerging pathogen and to identify mechanisms-related shifts in virulence, with the ultimate goal of mitigating the disease’s impact.

In order to compare the results obtained from different tools and to potentially contribute to the understanding of Bd biology, we analyzed the published data and looked for evidence of selective sweeps. We downloaded the SNVs for each isolate, gene annotation and published results [47]. After reformatting the data (gd_genotype), we uploaded it to Galaxy. Our first experiment was to check that our tools for identifying GO categories from a set of genes produce results comparable to the published results (the authors used custom computer scripts, which they make freely available). For the genes reported to have dN > dS (protein-coding differences between GPL and the outgroup isolate UM142), we found that GO terms with a significant enrichment included “DNA binding”, “protein binding”, “ATP binding”, and “nucleic acid binding”. We also found that the terms “microtubule motor activity” (*p =* 0.012), “microtubule-based movement” (*p* = 0.026), and “helicase activity” (*p =* 0.020) were enriched in these genes. The 35 GO terms significantly enriched for genes with LOH included “superoxide dismutase activity”, “oxidoreductase activity”, “oxidation-reduction process”, and “extracellular region”, while the 16 GO terms significantly depleted for genes with LOH include “chitin binding”. Finally, three GO terms were found to be significantly enriched for genes associated with CNVs: “aspartic-type endopeptidase activity”, “serine-type peptidase activity” and “proteolysis”. These results fit well with those in the original publication.

To go somewhat beyond what was published, we analyzed GO categories of genes in regions of high *F*_ST_ between an outgroup clade (UM142 and CLFT024-02) and the GP clade, as well as between two “populations” within GPL identified by PCA and phylogenetic analyses. Those results are included in the Galaxy history that we make available.

In summary, our Galaxy tools replicate many of the published results about Bd [46] and add some new observations. Our results suggest a fast evolution of genes associated to motility and helicase activity in the GP fungi lineage, as well as in proteolysis-related genes. As previously suggested, the peptidase genes are potentially important in Bd infection of amphibian skin [46]. Additionally, the genes associated to motility (i.e., microtubule motor activity and microtubule-based movement) might mediate in the dispersion capabilities of the zoospores and increase virulence [48]. Our results also suggest possible selection over a region of the Bd genome that includes five tandem SCP PR-1-like genes (BDEG_04273 to BDEG_04277), which are categorized under the GO term “extracellular region”. This selection seems to occur in all or a portion of the GLP lineage. Interestingly, the same region was found to have a loss of heterozygosity relative to UM142 and the GP clade. It has been suggested that these genes are involved in the pathogenesis and virulence of plant and animals pathogens, and may play a role in spore penetration and modulation of the host defense response [49,50]. While these results should be treated with healthy skepticism, due to a variety of possible difficulties, such as the observed high rate of copy-number variations in the Bd chromosomes, they illustrate the kinds of tantalizing observations that can be made with our Galaxy tools.

### 7. Human disease data

Many studies have sequenced the genomes from both normal and diseased tissues of the same individual, and looked for differences that might be associated with that disease, such as germ-line alleles that affect disease susceptibility, or variations in tumors that affect response to therapies. The new Galaxy tools can facilitate such investigations, as we now illustrate.

Analysis of human exome data has recently identified mutations in signal transducer and activator of transcription 3 (*STAT3*) in large granular lymphocyte (LGL) leukemia [51]. Concurrent to these findings, our group has recently undertaken whole genome sequencing of three paired patient lymphocyte/saliva samples to look for these and other mutations. With Galaxy we are able to use simple filters applied to gd_snp files to identify potential somatic mutations. Examples of the filtering include finding SNPs with differing genotype calls between LGL and saliva, a quality score of 20 or greater for both genotypes and a minimum read depth of 8 reads in each sample. The SNPs can be further filtered to identify changes of a particular type, such as LOH or somatic mutations. Using a file of amino-acid variants caused by the SNPs, one can identify which of the SNPs leads to a predicted change in protein structure. In our case SIFT [52] is available in Galaxy and can be used for this purpose with the added benefit that additional output fields, such as allele frequencies and OMIM disease associations are appended, if selected.

Applying this protocol, *STAT3* mutations were discovered in two of the three patients that correspond to amino acid changes of D661V and D661Y in genome 1 and 2 respectively. Previous reports [53] demonstrate constitutive *STAT3* activation in all LGL leukemia samples, though one study [51] reported direct *STAT3* mutations in only 31 of 77 patients. For this reason, the third genome was selected from a list of patients known to lack mutations in exon 20 or 21 of *STAT3*. Applying the same filters and SIFT algorithm to the SNPs from this genome did not reveal any mutations in any exon of *STAT3*. We then converted the Ensembl transcripts extracted from SIFT to their canonical transcripts and retrieved KEGG pathways using the Get Pathways tools (#24). A quick examination revealed two altered transcripts in the Janus Kinase (JAK)/STAT signaling pathway. Both consisted of 3' UTR mutations in the interleukin 6 receptor (*IL6R*) and *CBL*. Of these two, only the *IL6R* alteration is predicted to be in proximity to a conserved miRNA binding site according to the TargetScan [54] miRNA Regulatory Sites track on the UCSC Genome Browser [55,56]. If this variant alters miRNA binding and leads to increased translation of the IL6R, this could be one mechanism leading to aberrant *STAT3* activation in those patients that do not demonstrate direct *STAT3* mutation.

### 8. Mitochondrial polymorphism

In studies aimed at estimating evolutionary relationships, but where it is infeasible to collect data from the full (nuclear) genome, an alternative is to sequence the mitochondrial genome, which is far smaller and occurs at much higher copy number per cell. Recent methods that further enrich the concentration of mitochondrial DNA [57,58] make it possible to sequence mitochondria from very degraded samples, such as those from museum specimens. Nevertheless, the resulting data can leave intervals of the mitochondrial genome unsequenced, or sequenced to such low coverage that the results are unreliable. We have added tools to Galaxy that can perform some basic analyses for such datasets.

**Figure 6:**
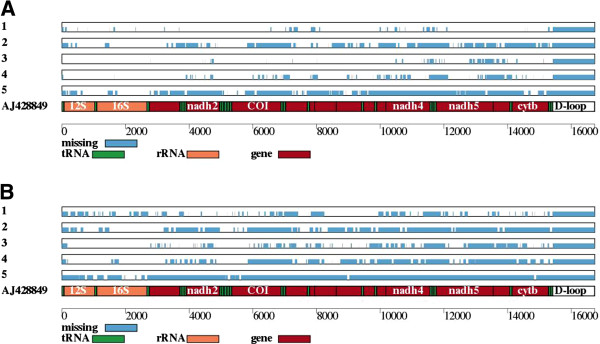
**Inadequately covered parts of colugo mitochondrial sequences.** Intervals with sequencing depth **(A)** below 5 and **(B)** below 30 for several Sunda colugos (*Galeopterus variegatus*), from a published study [57].

**Figure 7:**
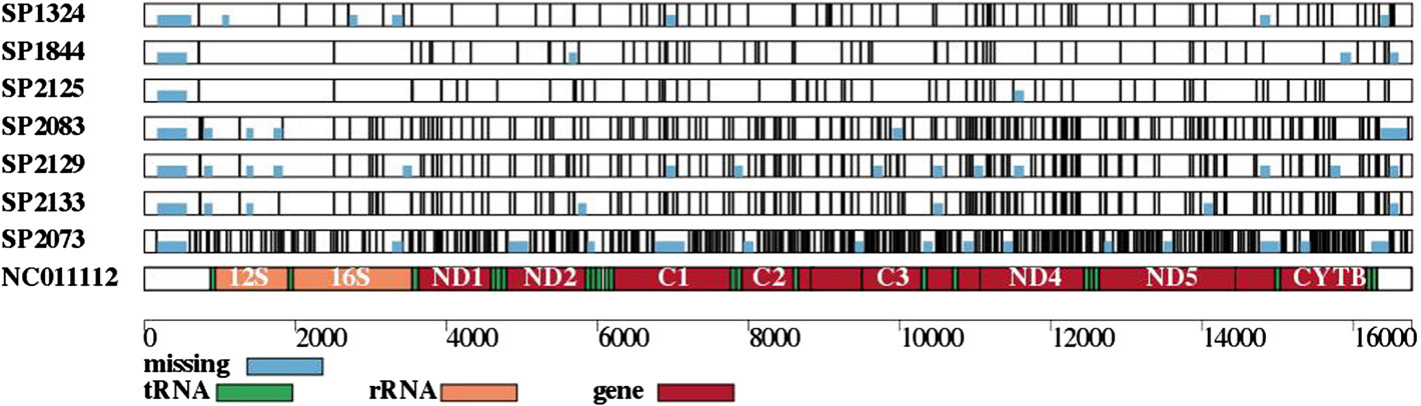
**Variants identified in cave-bear mitochondrial sequences.** Locations of SNVs (vertical lines) and unsequenced regions (blue rectangles) for several cave bears [58] relative to GenBank entry NC011112 [93]. SP1324, SP1844, SP2125 and NC01112 have been classified as one species of European cave bear (*Ursus ingressus*), and SP2083, SP2129 and SP2133 as another (*Ursus spelaeus*). SP2073 is an Asian cave bear (*Ursus deningeri kudarensis*).

SNVs in the mitochondrial genome can be represented in gd_snp or gd_genotype format. In addition, we abuse the gd_genotype format to store the sequence coverage at each position in the mitochondrial sequence. Thus the file might start as follows:

This indicates that the first sample has sequencing depth roughly 35 at the start of the mitochondrial sequence (column 5), while no reads from the second sample map there (column 6). We also include a file of gene annotations for the reference sequence, with lines like:

We provide several tools to process these files, including the production of a graphical representation of variants and/or the coverage depth (tools #33 and #34; Figures 6 and 7), computation of average pairwise difference (π; tool #35), and of a phylogenetic tree (tools #36 and #37). In each case, the user specifies a set of individuals and a minimum depth of coverage.

## Discussion

Regardless of how the polymorphism data are produced, using the Galaxy system to perform the analyses and to make the results available, can greatly facilitate reproducibility of the study by other groups, as well as apple-to-apple comparisons among data from different species. The published chicken sequence data described above provides a case in point – this study [31] sequenced pools, each containing DNA from multiple individuals, and so knowledge of numbers of reads corresponding to each allele, rather than a single genotype, was needed for each sample at each SNV. The authors attempted to provide relevant information in their dbSNP entries with a comment line telling in which samples the alleles were observed. However, they were aware that this information is not only difficult to extract from dbSNP, but it is also not sufficiently rich to allow their analyses to be reproduced; hence, the authors have contributed the appropriate data to Galaxy. More generally, having this and similar data sets on Galaxy substantially relieves the difficulties frequently encountered when attempting to reproduce published claims [59]. Moreover, reproducing published results is a prerequisite for exploring their resilience to changes in the parameters controlling the analysis; it is well-known that many computational tools produce output that is quite sensitive to input parameters, and progress in science is facilitated if readers can readily explore the brittleness of published claims.

The tools described in this paper were produced by members of a small genomics lab, an approach that had the benefit of relatively quick development, but drawbacks in terms of getting other groups to contribute their data. Being able to compare datasets with each other is tremendously valuable. For instance, as described above, we observed an average *F*_ST_ of 0.169 between the North and East populations of aye-aye, but without corresponding numbers from other data sets, it is unclear whether this value should be considered large or small. It would have been very helpful to have datasets provided by other groups, but thus far the only gd_snp and gd_genotype data sets are ones that we created or requested from the originating group. Unfortunately, biology journals are often lax about enforcing requirements that authors make their data readily available, and we have been unable to obtain certain requested datasets. Our belief is that if the toolset had been designed and contributed by a wider community, then those groups would be motivated to make their data available in Galaxy.

The development of Galaxy tools for the kinds of data discussed in this paper has only just begun. While short insertion/deletions are handled much like nucleotide substitutions, larger scale mutations, such as inversions, are not currently handled by our Galaxy tools, despite the fact that they are believed to sometimes directly influence phenotypic differences between populations [60,61]. However, proposed tools need to be carefully evaluated. Importantly, high computational demands are often a limiting factor; examples of tools whose extensive run times make them less desirable for incorporation into Galaxy include PSMC [62] for estimating ancestral population sizes, CoalHMM [63] for estimating population split times, and a tool for identifying a set of individuals that is an “optimal” choice for founding a captive-breeding population or a relocation effort [64]. Still, we hope the reader has become convinced that the resources we provide constitute a worthwhile addition to software for genome analysis.

## Methods

### Putting tools on Galaxy

Our analysis tools were integrated into the Galaxy web-based platform [1-4]. We followed the documentation [65] to install our own Galaxy instance. This instance was used as a test-bed for integrating our analysis tools. A tool definition file was written for each analysis tool, and all of these tool definition files were added to the tool-registry file as described on the Galaxy Wiki [66]. We added new data types [67] to our Galaxy instance to support our SNV table format. After verifying that all of our analysis tools were working as expected, we created a Galaxy Tool Shed [68] repository to share our tools with the Galaxy community by following the documentation [69], producing the final set of Genome Diversity tools [70].

### Tools for analyzing SNV tables

Phylogenetic trees can be determined when sequence data come from individuals (as opposed to pooled samples). For each pair of individuals, the “informative” SNVs having a user-specified read-coverage are used, and the distance between the two individuals is the number of genotypic differences divided by the number of informative sites. A neighbor-joining tree is constructed from these differences by QuickTree [71]. The output includes a viewable tree and formatted inputs to one of several popular tree-drawing tools. For small data sets, such as for a modest number of mitochondrial SNPs, we let users run RAxML [72]. Principal component analysis (tool #12) is preformed by *smartpca*[73], the ancestry analysis (#14) uses *admixture*[38,39] and the “Remarkable Intervals” tool implements a published algorithm [74], Figure 6. PCR primer sequences are computed off-line by Primer3 [75]. Determining an optimal set of breeding pairs (toll #22) is an instance of a classic computational problem known in the Operations Research literature as the “assignment problem”, and called “weighted optimal bipartite matching” by computer scientists; it can be solved efficiently [76].

The admixture tool (#15) uses allele frequencies in the source populations to estimate the probability that a genotype observed at one SNV in a potentially admixed individual would be generated by randomly sampling chromosomes in each of the three possible combinations (six combinations if there are three source populations). For instance, if the frequencies of the reference (or consensus) allele in the two source populations are *p* and *q*, then the probabilities of the admixed individual being homozygous for the reference allele are *p*^2^ if both chromosomes come from the first source population, *q*^2^ if both are from the second source population, and *p × q* if the individual has one chromosome from each source. The logarithms of these values are added along a chromosomal segment to estimate the (logarithm of the) probability that the sequence of genotypes along the segment would be produced. Logarithms are used so that values can be added instead of multiplied and to forestall underflow in computer arithmetic. A dynamic programming algorithm is used to select chromosomal positions where the source of the admixed segment is switched (e.g., from homozygous in the first source population to heterozygous).

### KEGG and Gene Ontology

We implemented a set of tools to evaluate the possible effect of mutations on phenotypic differences. The first group of tools assesses the over-representation of input genes in phenolic categories (i.e., GO terms and KEGG pathways), and the second uses network metrics to calculate the impact of these genes in a given phenotype (i.e., KEGG pathways).

GOs are a broadly used category of gene annotations that describe their functions through the use of domain-specific ontologies [77]. Each gene is associated to one or more GO terms, and in turn, each GO term can be associated to one or more genes. Our set of programs includes the Rank Terms tool (#27) to determine the enrichment of a gene list (i.e., mutated genes) in GO terms. To do so, each gene is associated to a GO term following the Ensembl annotation [78]. Further, the probability of GO term enrichment and depletion among the genes in the input list is calculated with a two-tailed Fisher exact test, as suggested [79]. The tool returns a table that ranks the GO terms based on the percentage of genes in an input dataset (out of the total in each category in a background list) and their enrichment/depletion probability.

Network-based approaches have been recently introduced with promising results to capture the intricate relation of genes, regulatory elements and phenotypes [80,81]. The Rank Pathways tool (#25) is designed to study phenotypes as networks. This tool takes, as input, the set of metabolic pathways and biological processes in the KEGG database [82,83] and ranks them based on two criteria. The first criterion returns a table that ranks the KEGG pathway based on the percentage of genes in an input dataset (out of the total in each pathway) and their enrichment/depletion probability (calculated by a two-tailed Fisher exact test).

The second ranking criterion ranks KEGG pathways based on the change in length and number of paths connecting sources and sinks between pathways that exclude or include the nodes representing the genes in an input list. Sources are all the nodes representing the initial reactants/products in the pathway. Sinks are all the nodes representing the final reactants/products in the pathway. In detail, the mean length and number of paths between sources and sinks is calculated for each pathway including and excluding the genes in the input dataset; further, the change in both parameters is estimated and ranked [84,85]. Gene names and networks are obtained from each KGML pathway file from the KEGG database of the reference species.

In addition, the Get Pathways tool (#24) maps KEGG genes and pathways to Ensembl codes, while the Pathway Image tool (#26) plots KEGG pathways highlighting genes of interest respectively (e.g., Figure 3). In more detail, the second tool takes as input datasets with KEGG gene codes and pathways, links the genes present in the input table to specific modules (i.e., a collection of functional units) and returns an image of a KEGG pathway highlighting (in red) the modules representing genes in the input dataset.

### Chicken

The published SNVs [31] were kindly provided to us by Carl-Johan Rubin and Leif Andersson. Importantly, the sequences were from pooled samples of birds, so the numbers of reads observed for each allele in each sample (rather than just a “genotype” for the sample) was required to reproduce their results. We created a Galaxy gd_snp table. For each sample, in addition to the two allele counts, the SNV had a “genotype” that we extracted from comments in the dbSNP records listing the samples where each allele was observed, which we included to permit attempts to reproduce some of the published [31] using just the information in dbSNP. (We were unable to accomplish this feat). Extracting that information required help from the dbSNP staff at NCBI. Since no quality values were available to us, we used the place-holder “–1” in columns 5, 9, 13-45. The data and a command “workflow” for the results described in this paper are available on Galaxy.

### A southern African genome

Three methods were applied to detect admixtured haplotype blocks in a southern Bantu genome (ABT): PCAdmix [15], HAPMIX [14], and the Galaxy admixture tool. Applying those methods required population datasets of two or three putative ancestral populations in order to assign ancestries to each SNV or particular size of haplotype. We retrieved genotyping SNV datasets of various populations from two human variation projects, HGDP [86] and HapMap [87], and one publication [88]. We selected 419,974 SNVs that were common among the datasets, after filtering out multiple-allelic and possible “flipped” SNVs. For the estimation of ancestry of the Bantu individual, Khoisan, Bantu, Yoruba, and two non-African (CEU and CHB) populations were selected from the datasets. For the accuracy of analyses, we included only unrelated individuals and excluded outlier individuals, which were not clustered with the corresponding populations in the PCA analysis. The final dataset used in this study consisted of 419,974 SNVs from 481 individuals. Regarding ABT, we extracted the genotypes of the same SNV positions from the ABT genome sequences [37].

### Pig

The table of porcine SNVs was contributed by Martien Groenen.

### Chytrid fungus

We converted the table Bd_49.selectedSNPs.5.ACGT.10X.tab [47] to gd_genotype format. From the same website we obtained a mapping of gene names to GO categories, and lists of genes with dN > dS, with LOH, and with CNVs. We extracted gene annotations from the file batrachochytrium_dendrobatidis_1_genome_summary_per_gene.txt[89].

### Mitochondria

The coverage data for colugo were contributed by William Murphy.

## Availability of supporting data

The data sets and tools sufficient to reproduce results described in this paper are available on the GigaGalaxy website [90]. The tools discussed here are available from the Galaxy website [1], under “Genome Diversity”. Links to the materials and future examples will also be made available from a Galaxy page [91], along with documentation for using the tools [92]. Please send requests for other materials to webb@bx.psu.edu.

## Abbreviations

AD: All domestic (chickens); also abbreviates “Allelic Depth” in VCF file; AIM: Ancestry informative marker; Bp: Base pair; CNV: Copy Number Variation; gd: Genome diversity; GO: Gene ontology; GPL: Global Panzootic Lineage; KEGG: Kyoto Encyclopedia of Genes and Genomes; LOH: Loss of Heterozygosity; LSBL: Locus-specific branch length; PCA: Principal components analysis; QTL: Quantitative Trait Locus; SNP: Single-nucleotide polymorphism; SNV: Single-nucleotide variant; YNP: Yellowstone National Park (wolves).

## Competing interests

The authors declare that they have no competing interests.

## Authors’ contributions

The project was designed by WM, with guidance from SCS and GHP. The Galaxy tools were written, installed, and documented by OCB-R, AR, RB, BG, CR and WM. QL provided statistical expertise. The paper was written by WM, with sections contributed by OCB-R, HLK, TLO, TPL and BMH. All authors read and approved the final manuscript.
